# Single-Cell RNA-Seq Analysis of Olfactory Mucosal Cells of Alzheimer’s Disease Patients

**DOI:** 10.3390/cells11040676

**Published:** 2022-02-15

**Authors:** Riikka Lampinen, Mohammad Feroze Fazaludeen, Simone Avesani, Tiit Örd, Elina Penttilä, Juha-Matti Lehtola, Toni Saari, Sanna Hannonen, Liudmila Saveleva, Emma Kaartinen, Francisco Fernández Acosta, Marcela Cruz-Haces, Heikki Löppönen, Alan Mackay-Sim, Minna U. Kaikkonen, Anne M. Koivisto, Tarja Malm, Anthony R. White, Rosalba Giugno, Sweelin Chew, Katja M. Kanninen

**Affiliations:** 1 A. I. Virtanen Institute for Molecular Sciences, University of Eastern Finland, 70210 Kuopio, Finland; riikka.lampinen@uef.fi (R.L.); feroze.fazaludeen@uef.fi (M.F.F.); tiit.ord@uef.fi (T.Ö.); liudmila.saveleva@uef.fi (L.S.); emma.kaartinen@uef.fi (E.K.); fdezacosta98@gmail.com (F.F.A.); marcela.cruzhaces@gmail.com (M.C.-H.); minna.kaikkonen@uef.fi (M.U.K.); tarja.malm@uef.fi (T.M.); sweelin.chew@gmail.com (S.C.); 2 Department of Computer Science, University of Verona, 37134 Verona, Italy; simone.avesani@univr.it (S.A.); rosalba.giugno@univr.it (R.G.); 3 Department of Otorhinolaryngology, University of Eastern Finland, Kuopio University Hospital, 70210 Kuopio, Finland; elina.penttila@kuh.fi (E.P.); heikki.lopponen@kuh.fi (H.L.); 4 Brain Research Unit, Department of Neurology, School of Medicine, University of Eastern Finland, 70210 Kuopio, Finland; juha-matti.lehtola@uef.fi (J.-M.L.); toni.saari@uef.fi (T.S.); sanna.hannonen@kuh.fi (S.H.); anne.koivisto@kuh.fi (A.M.K.); 5 Department of Neurology, Neuro Centre, Kuopio University Hospital, 70210 Kuopio, Finland; 6 Griffith Institute for Drug Discovery, Griffith University, Nathan, QLD 4111, Australia; a.mackay-sim@griffith.edu.au; 7 Department of Neurology and Geriatrics, Helsinki University Hospital and Neurosciences, Faculty of Medicine, University of Helsinki, 00014 Helsinki, Finland; 8 Department of Cell and Molecular Biology, QIMR Berghofer, Medical Research Institute, Mental Health Program, Herston, QLD 4006, Australia; tony.white@qimrberghofer.edu.au

**Keywords:** Alzheimer’s disease, olfactory mucosa, amyloid beta, single-cell RNA-sequencing, mitochondria

## Abstract

Olfaction is orchestrated by olfactory mucosal cells located in the upper nasal cavity. Olfactory dysfunction manifests early in several neurodegenerative disorders including Alzheimer’s disease, however, disease-related alterations to the olfactory mucosal cells remain poorly described. The aim of this study was to evaluate the olfactory mucosa differences between cognitively healthy individuals and Alzheimer’s disease patients. We report increased amyloid-beta secretion in Alzheimer’s disease olfactory mucosal cells and detail cell-type-specific gene expression patterns, unveiling 240 differentially expressed disease-associated genes compared to the cognitively healthy controls, and five distinct cell populations. Overall, alterations of RNA and protein metabolism, inflammatory processes, and signal transduction were observed in multiple cell populations, suggesting their role in Alzheimer’s disease-related olfactory mucosa pathophysiology. Furthermore, the single-cell RNA-sequencing proposed alterations in gene expression of mitochondrially located genes in AD OM cells, which were verified by functional assays, demonstrating altered mitochondrial respiration and a reduction of ATP production. Our results reveal disease-related changes of olfactory mucosal cells in Alzheimer’s disease and demonstrate the utility of single-cell RNA sequencing data for investigating molecular and cellular mechanisms associated with the disease.

## 1. Introduction

The olfactory mucosa (OM) is critical for the sense of smell and in direct contact with the brain [1]. The OM is composed of three primary components, the epithelium, basement membrane, and lamina propria. In vivo, the healthy human OM consists of several cell types, including olfactory sensory neurons, basal cells (stem cells), Bowman’s gland cells, vascular smooth muscle cells, sustentacular cells, and immune cells. The cells of the human OM can be obtained for studies with a relatively non-invasive biopsy of the nasal septum taken under local anesthetic [2].

Anosmia, loss of the sense of smell, has been linked to the early phases of several neurodegenerative diseases including Alzheimer’s disease (AD) [1,3,4,5]. Nasal secretions of patients with late-onset AD contain elevated amounts of amyloid-beta (Aβ) and phosphorylated tau [6,7,8], indicative of pathological processes occurring in the nasal cavity. Postmortem histological analysis of the AD OM has revealed immunostaining for filamentous tau in cells close to the OM basal membrane [9]. Intracellular Aβ and increased Aβ aggregation in the apical surface of the epithelium have also been found in histological sections of OM biopsies derived from living anosmic patients with late-onset AD [10]. However, little information exists on cell-type-specific, AD-related alterations to specific populations of OM cells. The only reported cell-type-specific observations thus far are from olfactory neurons in postmortem analyses of AD tissues [11,12], and from OM-derived neuroblast cultures that exhibit elevated oxidative stress and altered amyloid precursor protein (APP) processing [13,14]. Single-cell RNA sequencing (scRNA-seq) has been used to characterize cells obtained from other tissues of AD patients, such as the postmortem brain [15,16,17,18], and this technology has revealed important insight into the pathology. ScRNA-seq is a powerful technology that enables the elucidation of genetic heterogeneity [19] and is very crucial for defining cell states and types from data, revealing marker genes, and identifying developmental trajectories that relate cells to cells [20]. Most importantly, high-resolution analysis enables the discovery of cellular differences [19,20,21,22], strengthening this has been the continual development of computational methods for data processing, analysis, and integration methods [20]. In contrast, single-cell proteomics/metabolomics is a field still in its early stages of development (review [23,24,25]). Recent scRNA-seq studies have revealed the cellular content of the biopsied OM of healthy individuals [26] and transcriptomic changes in OM cells in presbyosmia, an olfactory loss related to aging [27].

In this study, we harvested OM biopsies from cognitively healthy individuals and age-matched patients with late-onset AD to profile disease-linked functional alterations and changes in gene expression at the single-cell level. Additionally, comparing our scRNA-seq data with the entorhinal cortex, AD scRNA-seq data had eight common DEGs between the control and AD groups. The presence of these common DEGs underscores the importance of these genes in AD regardless of the tissue type assessed and suggests that the entorhinal cortex and OM, both are vulnerable to the pathogenesis of early AD, exhibit disease-specific alterations. Together, these results support the utility of the OM as a physiological in vitro model of AD and provide a unique cellular-level view of transcriptional alterations associated with the disease pathology.

## 2. Materials and Methods

### 2.1. Ethical Considerations

The study was performed with the approval of the Research Ethics Committee of the Northern Savo Hospital District (permit number 536/2017). The oral and written information concerning the study was provided by the study investigator or study nurse. The written informed consent was collected from all the voluntary study participants and proxy consent from the family members/legally acceptable representatives of persons with mild Alzheimer’s type dementia.

### 2.2. Patients and OM Cell Cultures

A total of 12 voluntary study patients with AD type mild (CDR 1) dementia were recruited via the Brain Research Unit, Department of Neurology, University of Eastern Finland. AD diagnostic examinations had been carried out at Brain Research Unit or at the Department of Neurology, Kuopio University Hospital prior to study recruitment. All the patients with AD dementia fulfilled the NIA-AA clinical criteria of progressive AD and MRI imaging or FDG-PET study had showed degenerative process or, in CSF biomarker examination, was found biomarker (beta-amyloid, tau, and phos-tau) changes typical to AD [28]. Eleven cognitively healthy control subjects were recruited via the Department of Otorhinolaryngology, Kuopio University Hospital, Finland from patients undergoing a dacryocystorhinostomy (DCR) surgery, or from the already existing registries of the Brain Research Unit of the University of Eastern Finland. The cognition of all the study’s patients was evaluated utilizing the Consortium to Establish a Registry for Alzheimer’s Disease (CERAD) neuropsychological battery [29,30]. The sample sizes of the study groups were not predetermined. The age of the patients with AD and cognitively healthy control subjects were on average 68.3 years and 70.6 years, respectively. For the AD group, 50% of the study subjects were males and 50% females, whereas for control subjects 27.3% were males and 72.7% females. A venous blood sample was taken from all study participants for use in *APOE*-genotyping. Based on genotyping, 66.7% of the patients with AD and 36.4% of cognitively healthy control subjects had at least one *APOE* ε4 allele. *APOE*-genotyping of the study subjects was performed as described previously [31]. The polymerase chain reaction and following allelic discrimination were performed on QuantStudio 5 Real-Time PCR System platform (Applied Biosystems, Waltham, MA, USA). Patients and cognitively healthy study participants were tested for their sense of smell for 12 odors (Sniffin’ Sticks, Heinrich Burghart GmbH, Wedel, Germany) and classified as normal, hyposmic, or anosmic [32]. For the control group, 63.6% of the study subjects had a normal sense of smell and the remaining 36.4% had hyposmia. For the AD group, equal numbers of patients (41.7%) had normal olfactory function or hyposmia. In addition, 16.7% of the patients with AD were classified as anosmic.

A piece of the OM was collected as a biopsy from the nasal septum, close to the roof of the nasal cavity as previously described [2,33]. Primary OM cell cultures were established according to the published protocol [2,33] with small modifications. In short, any remaining blood or cartilage was removed under a dissection microscope and the flattened tissue piece was rinsed several times with cold Hank’s Balanced Salt Solution. To separate olfactory epithelium and underlying lamina propria, the tissue was enzymatically digested for 45 min with dispase II as 2.4 U/mL (Roche, Basel, Switzerland) followed by further digesting the lamina propria with DMEM/F12 media containing 0.25 mg/mL collagenase H (Sigma-Aldrich, St. Louis, MO, USA) for up to 10 min. Finally, the digested olfactory epithelium and lamina propria were combined and seeded on poly-D-lysine (Sigma-Aldrich) coated 6-well in order to let the cells migrate out of the tissue pieces and proliferate in growth medium at 37°C, 5% CO2. Half of the culture media was changed every 2–3 days for a total of 8 to 19 days before passaging the cultures and freezing the primary cell lines in liquid nitrogen for later use in a solution containing 90% heat-inactivated FBS and 10% dimethyl sulfoxide. Cells in primary passages of 2–3 were used for scRNA-seq and primary passages 4–6 for biochemical analyses.

### 2.3. Enzyme-Linked Immunosorbent Assays

The culture medium was collected, and cells were lysed in RIPA buffer supplemented with a 1x cOmplete protease inhibitor cocktail (Roche, Basel, Switzerland). Cell lysates and medium for phosphorylated tau and total tau Enzyme-linked immunosorbent assays (ELISA) were collected in RIPA buffer supplemented with a 1x cOmplete protease inhibitor cocktail as well as phosphatase inhibitor cocktail 2 (#P5726, 1:1000, Sigma-Aldrich, MO, USA). Samples were stored at −70°C until analysis. Fifty microliters of each medium, cell lysate or plasma sample, was analyzed in singlets for cell lysates and media samples, and in duplicates for plasma samples using ELISA kits (all from Invitrogen, Waltham, MA, USA) for human Amyloid beta 40 (#KHB3481), Amyloid beta 42 (#KHB3544), phosphorylated tau, pT181) (#KHO0631) and tau (total) (#KHB0041), according to the manufacturer’s instructions. Total protein amounts of the OM cell lysates were quantified with BCA assay (Pierce™ BCA Protein Assay Kit, Thermo Scientific, Waltham, MA, USA) according to the manufacturer’s instructions. The results were calculated as pg/mg protein ± SD for the OM cells and for plasma samples as pg/mL plasma ± SD.

### 2.4. Cell Hashing and Single Cell RNA Sequencing

Cells from three cognitively healthy control lines (average age 71.7 years) and five AD patients (average age 67.2 years), all females, were harvested from a culture with TrypLE Express (Gibco, Waltham, MA, USA) and resuspended in PBS containing 2% BSA. TotalSeq™ -A anti-human Hashtag Antibodies (BioLegend, San Diego, CA, USA) were used according to the producer’s instructions to stain the cells of individual donors before pooling the cells as one pool for control subjects and two pools for AD patients to have cells from two to three individuals in one pool. Equal numbers of cells from each donor were pooled together. Next, the cell pools were filtered through a 30 µm strainer, and the cells were resuspended in PBS containing 0.04% BSA. The viability of the single-cell suspension pools was >90% based on Trypan blue staining.

The sample pools were run on a Chromium Chip B with the Chromium Single Cell 3′ Library and Gel Bead Kit v3 kit (10x Genomics, CA, USA) according to the manufacturer’s protocol with a targeted cell recovery of 10,000 cells per lane. In the cDNA amplification step, the cell hashing protocol from New York Genome Center Technology Innovation Lab (version 2019-02-13, New York, NY, USA) was followed (https://genomebiology.biomedcentral.com/articles/10.1186/s13059-018-1603-1, 27 June 2019). The hashtag oligonucleotide primer (HTO primer 5’GTGACTGGAGTTCAGACGTGTGCTC’3) was added to the cDNA amplification mix. During cDNA cleanup, the supernatant contained the HTO-derived cDNAs (<180 bp) and the pellet of mRNA-derived cDNAs (>300 bp). The mRNA-derived cDNAs were processed according to the protocol provided by 10x Genomics and the HTO-derived cDNAs fraction was processed according to the cell hashing protocol referenced above. The 3’ gene expression libraries and HTO sequencing libraries were pooled and sequenced at an approximate depth of 50,000 reads per cell for the 3’ gene expression libraries and 5000 reads for HTO libraries using the NovaSeq S1 (Illumina, San Diego, CA, USA) flow cells.

### 2.5. Quality Control and Downstream Analysis

Cell Ranger v.3.0.2 was used to analyze the raw base call files. FASTQ files and raw gene-barcode matrices were generated using the command ‘mkfastq’ and the ‘count’ command, respectively, and aligned human genome GRCh37 (hg19). The samples were integrated into R v.4.0.3 and the three generated Seurat objects—two related to AD samples and the other to control samples—were analyzed using the Seurat package v.4.0.3 to perform downstream analysis, clustering of the cells, and differential expression [34]. A more detailed description of the analysis can be found in Appendix A. The top 30 most up-regulated and significant differentially expressed genes between the clusters (Table S1) were inputted into EnrichR [35,36,37] and HumanBase [38] tools, using Gene Ontology and cellular pathway information to confirm the cell-type annotation. To be more confident of the final cell-type annotation, we also considered the effects of cell cycle heterogeneity in our data calculating the cell cycle phase score for each cell.

### 2.6. Differential Expression and Enrichment

We performed the differential expression analysis between all the cells of the AD and control libraries, and between these two groups by cell type. To understand which biological processes are perturbed by different cell conditions, enrichment analysis was performed using a non-parametric unsupervised method called Gene Set Variation Analysis (GSVA, v. 1.36.3, open-source software) [39] and the open-source pathway database Reactome (1 February 2022) [40,41]. Reactome web tool was also used to compare GBC-like cells with those annotated by Durante et al. [26]. See more details of the analysis in Appendix A.

### 2.7. Analysis of Mitochondrial Respiration

The MitoStress test was performed according to the manufacturer’s instructions (Agilent, Santa Clara, CA, USA). The OM cells were seeded on cell XFe96-well culture microplates two days before the assay. The Seahorse XF DMEM medium, pH 7.4 (#103575-100, Agilent), was supplemented with 25 mM D-glucose (Sigma-Aldrich), 2 mM sodium pyruvate (Gibco), 2 mM GlutaMAX (Thermo Fisher Scientific, Waltham, MA, USA). The oxygen consumption rates (OCR) following injections with oligomycin, FCCP, rotenone, and antimycin A (all at a final concentration of 1 μM, Sigma-Aldrich) were recorded with Seahorse XFe96 Analyzer (Agilent). The total protein concentrations/well were measured with Pierce BCA protein assay kit (Thermo Fisher Scientific) from cells lysed in 1 × RIPA buffer and the data were analyzed utilizing Wave 2.6.0 (Agilent, Santa Clara, CA, USA). The data were combined from three individual experiments where each cell line was included in one of the experiments.

### 2.8. Quantification of the Intracellular Levels of ATP

Quantification of the intracellular ATP levels of OM cells was performed using the ATPLite Luminescence Assay System (Perkin Elmer, Waltham, MA, USA) according to the manufacturer’s instructions. The cells were seeded on 48-well plates as 40,000 cells/well two days prior to the assay. The Wallac Victor 1420 microplate reader (Perkin Elmer) was used to read the luminescence.

### 2.9. Western Blotting

Cells were lysed and collected in 1 × Laemmli buffer (62.5 mM Tris-HCl (pH 6.8), 2.3% SDS, 5% β-mercaptoethanol, 10% glycerol, 0.02% bromophenol blue) and ran on 7.5% SDS-PAGE Tris-glycine gels. After transferring the proteins to PVDF membranes with the Trans-Blot Turbo Transfer System (Bio-Rad, Hercules, CA, USA), the membranes were blocked with 5% non-fat dry milk solution prepared in 0.2% Tween-20/0.01 M PBS for 30 min. The immunodetection of LRP1 was performed overnight at 4 °C with the anti-LRP1 antibody (Cell Signaling Technology Inc., Danvers, MA, USA, 26387, 1:1000). Anti-β-actin antibody (Sigma-Aldrich A5441, 1:5000) was used for loading control. For detection of the LRP1 and β-actin, the membranes were incubated for 2 h at room temperature in HRP conjugated IgG anti-rabbit secondary antibody (Bio-Rad 170-65-15, 1:3000) or Cy5 conjugated IgG anti-mouse secondary antibody (Jackson ImmunoResearch Laboratories Europe Ltd., Cambridgeshire, UK 715-175-151, 1:1000 dilution). A detection technique based on enhanced chemiluminescence (SuperSignal™ West Pico PLUS Chemiluminescent Substrate, Thermo Fisher Scientific) was used for developing the membranes incubated with the HRP conjugated secondary antibody. All membranes were imaged utilizing the Bio-Rad ChemiDoc XRS + System.

### 2.10. Statistical Methods and Graphical Illustrations

The GraphPad Prism 8.1.0 (GraphPad Software Inc. San Diego, CA, USA) software was used for statistical analysis of the data. Mean values in ELISA and CBA analyses, between control and AD, were compared using unpaired two-tailed t-test with or without Welch’s correction or with two-way ANOVA. Error bars in the figure legends 1-4 represent standard deviation (SD), except for Figure 4b where the error bars for OCR values are shown as the standard error of the mean (SEM). Statistical significance was assumed for *p*-values < 0.05. The graphical illustrations were created with BioRender.com and the open-source vector graphics editor Inkscape 0.91.

## 3. Results

### 3.1. Secretion of Aβ_1–42_ Is Increased in AD OM Cells

**Figure 1:**
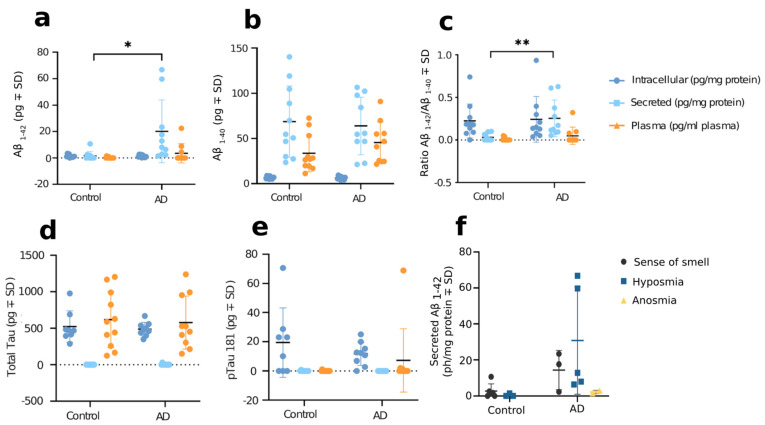
Aβ secretion is increased in AD OM cells. OM cells harvested from biopsies were plated cultured for 7 days prior to collection of culture media and cell lysates for ELISA assays. The results were normalized to the total amount of protein measured from cell lysates. (**a**) ELISA assay for Aβ_1–42_. (**b**) ELISA assay for Aβ_1–40_. (**c**) Ratio of Aβ_1–42_ over Aβ_1–40_. In Aβ ELISA assays, *n* = 11 for cognitively healthy controls and *n* = 10 for Alzheimer’s disease patients. (**d**) ELISA assay for total tau. (**e**) ELISA assay for P181-tau. In tau ELISA assays, *n* = 8 for cognitively healthy controls and *n* = 9 for AD patients. Quantification of secreted and intracellular Aβ_1–42_, Aβ_1–40_, tau, and P181-tau between control and AD OM cells were performed with t test (unpaired, two-tailed, Welch’s correction 2-way ANOVA with Tukey multiple test). (**a**) * *p* < 0.05. (**c**) ** *p* < 0.01. For all graphs, data are presented as mean ± SD and calculated the statistics as a difference in means ± SEM. (**f**) The levels of secreted Aβ_1–42_ were not found to correlate to the status of the donor’s sense of smell. OM cells harvested from biopsies were cultured for 7 days prior to the ELISA assay, assessing levels of Aβ_1–42_ in media collected from OM cells. The results were normalized to the total amount of protein measured from cell lysates and then separated into subgroups based on the sense of smell status of the biopsy donor.

To test whether OM cells obtained from AD donors exhibit the typical pathological hallmarks of the disease, we first analyzed intracellular and secreted levels of Aβ_1–42_, Aβ_1–40,_ total tau, and tau phosphorylated at threonine 181 (P181-tau) in cognitively healthy individuals and AD patients using ELISA (Figure 1). A significant increase of secreted Aβ_1–42_ was observed in AD OM cells (18.52 ± 7.590 pg/mg protein, *p* = 0.0366) when compared to cells of cognitively healthy individuals. In addition, the ratio of secreted Aβ_1–42_ over Aβ_1–40_ was higher in AD OM cells in comparison to controls (0.2230 ± 0.06919, *p* = 0.0096). No difference was observed in levels of Aβ_1–40_, total tau, or P181-tau. Likewise, these proteins remained unaltered in the plasma of AD patients. The levels of secreted Aβ_1–42_ did not correlate to the status of the donor’s sense of smell, however, both anosmic and hyposmic individuals were not included in all experimental groups (Figure 1f). Nevertheless, the OM cells of the AD patients with hyposmia secreted notably increased amounts of Aβ_1–42_ (30.42 ± 13.34 pg/mg protein, *p* = 0.0847) in comparison to the cognitively healthy individuals with hyposmia. There was no significant difference in the secretion Aβ_1–42_ from OM cells obtained from donors with at least one *APOE* ε4 allele when compared to donors with only *APOE* ε3 alleles (Figure S1).

### 3.2. Diversity of the Human OM Cells in Health and AD

**Figure 2:**
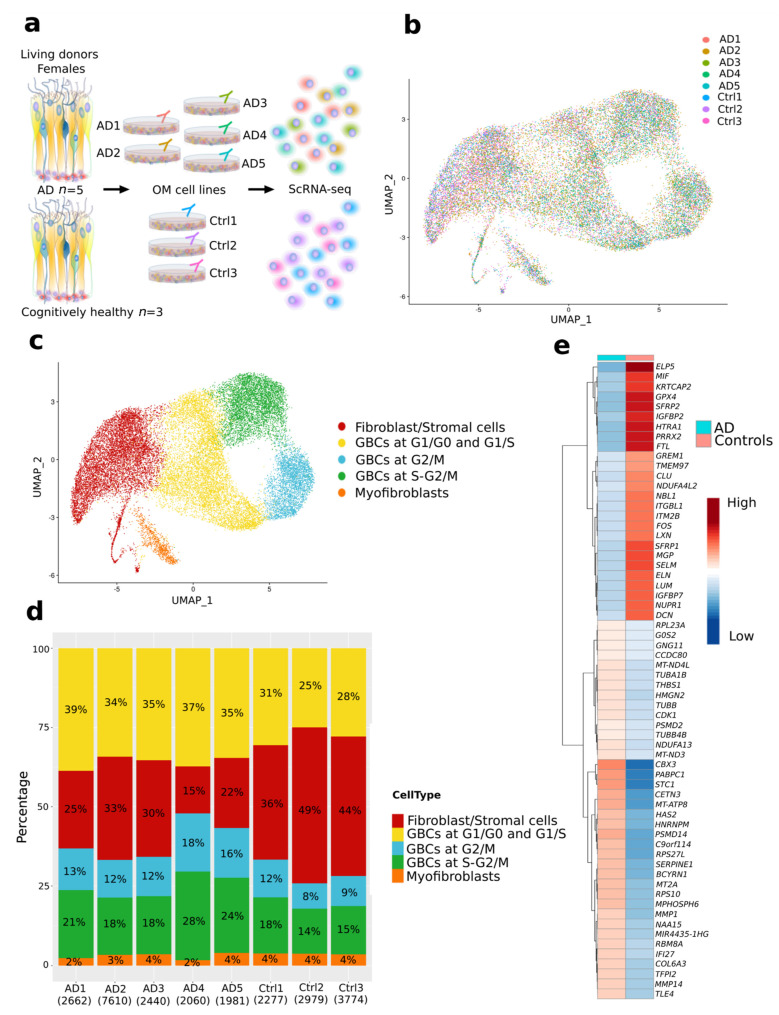
ScRNA-seq data revealed AD-specific alterations in OM cells. (**a**) Schematic illustration of the sample material and scRNA-seq workflow. (**b**) UMAP visualization of the clustering of single cells and showing by colors the cells derived from individual cognitively healthy donors or patients with AD. (**c**) Annotated cell types in samples derived from cognitively healthy individuals and patients with AD. (**d**) Proportion (%) of the cells of the total samples derived from each cell type. Under each sample, ID shows the total number of cells for each sample (**e**) Heatmap depicting a subset of the AD-related genes differentially expressed between AD and control libraries. The data are presented as the average of the log normalized and scaled expressions of the most differentially expressed genes (avg(log2FC) < −0.4, avg(log2FC) > 0.4) in AD and control cells.

To investigate the cell type diversity and changes in AD OM cells, we performed scRNA-seq of OM cultures derived from cognitively healthy controls and patients with AD (Figure 2a). To reduce variability, OM cell lines from female donors (age average 68.9) were used for scRNA-seq.

We sequenced 10,816 live cells for the control library, and 12,582 and 8885 live cells for the two AD libraries. The median number of genes per cell in the control and two AD libraries were 4066, 3336, and 4450, respectively. After quality-control filtering, 9030 cells for controls and 16,753 cells for AD were obtained (Figure 2b and Figure S2). Single-cell transcriptomes were clustered in uniform manifold approximation and projection (UMAP) to assess cellular heterogeneity with the Seurat clustering combined with EnrichR and HumanBase tools, and data obtained from Durante et al. [26]. After data integration, five clusters were identified, consisting of fibroblast/stromal-like cells, globose basal cell (GBC) -like cells at different cell cycle phases and under differentiation, and myofibroblast-like cells (Figure 2b,c and Figure S3, Table S2). Figure 2d and Table S3 show the proportion of cell types present in the OM cultures of each donor. By proportion, the largest number of cells in the OM cultures were annotated as GBC-like and fibroblast/stromal cell-like cells. In AD the proportion of all GBC-like cells was slightly higher, approximately 72%, from the total number of cells/donors, compared to OM cells derived from cognitively healthy individuals where the proportion of all GBC-like cells ranged from 47.1% to 60.1%. A small proportion of cells was annotated as myofibroblast-like cells in both libraries (Figure 2d, Table S3). Information of the annotated cell types (fibroblast/stromal-like cells, GBC-like cells, myofibroblast-like cells) was used to characterize AD-related gene expression perturbations occurring in the OM on the level of single cells. We identified a total of 76 identical Reactome pathways between GBC cells (all cell cycle stages considered as one group of GBCs) in this study, and the GBCs in vivo from the data by Durante et al. [26]. The pathways for this comparison were derived based on the most enriched genes for those cell clusters and the overlapping significance was checked through a multi-set intersection statistical test [42] (Figure S5). Among the identical pathways, there are seven pathways related to the neuronal system, two for developmental biology and ten for signal transduction (Table S4).

### 3.3. Differential Expression Analysis Reveals AD-Related Alterations of Gene Expression in the OM Cells

The single-cell transcriptomic analysis of OM cells revealed a total of 240 AD-associated genes differentially expressed between the OM cell libraries of cognitively healthy control individuals and patients with AD (Table S5). Of these DEGs, 144 were up-regulated and 95 down-regulated in the AD OM cells. Figure 2e illustrates a subset of the most differentially expressed (avg(log2FC) < −0.4, avg(log2FC) > 0.4) AD-associated genes between the OM libraries. *STC1*, *BCYRN1*, *CBX3*, *TFPI2*, *MMP1*, *PABPC1*, *MT-ATP8,* and *RPS10* were the top eight up-regulated DEGs, and *SFRP2*, *MGP*, *IGFBP2*, *HTRA1*, *PRRX2*, *COL3A1*, *ELP5,* and *SFRP1* formed the top eight most down-regulated DEGs.

### 3.4. Analysis of Individual Cell Types Reveals Distinctive AD-Associated Genes and Pathways

**Figure 3:**
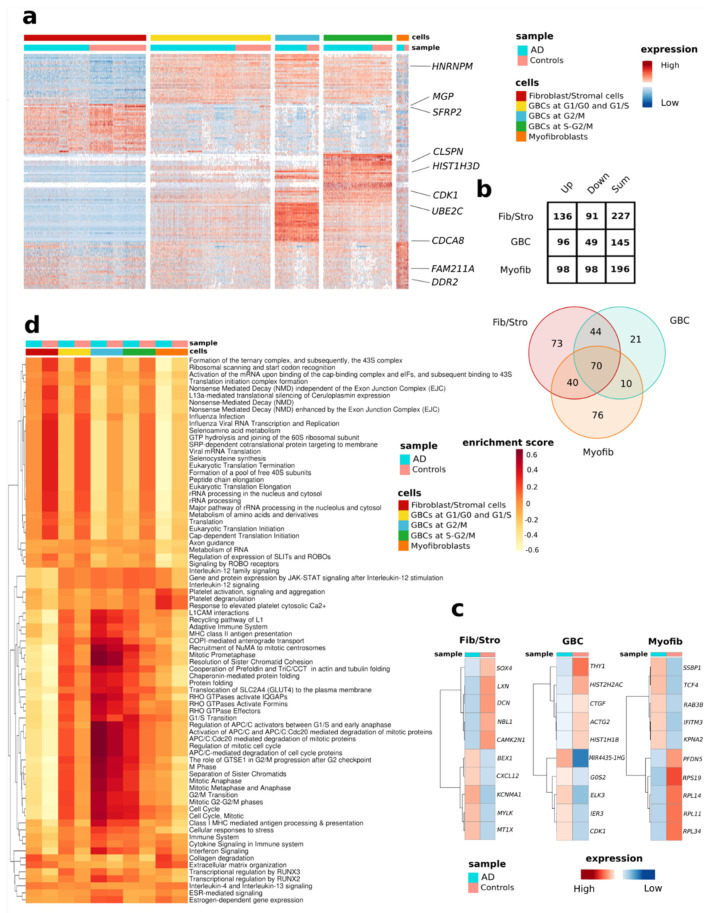
Cell-type-specific pathways altered in AD. (**a**) Heatmap depicting differential gene expression at the single-cell level for the most differentially expressed genes for each annotated cell type separated between donors with AD and cognitively healthy individuals. Each expression of the values is scaled and centered in 0. The range of expression values is from −4 to 4. (**b**) Numbers of differentially expressed AD-related genes by cell types common for controls and AD shown as table and Venn diagram. (**c**) The top five up-regulated and down-regulated AD-related genes differentially expressed between control and AD OM by cell types. The expression range is scaled between 1 and −1. (**d**) Heatmap of the subset of differentially expressed pathways for cell types common between AD and controls shows GSVA enrichment score of the pathways obtained for the DEGs with adjusted *p*-value < 0.05. The enrichment score range is from −0.6 to 0.6. (GBC, globose basal cell-like cells). Fib/Stro, fibroblast/stromal -like cells. Myofib, myofibroblast-like cells. OCR, oxygen consumption rate.

In contrast to comparing overall transcriptomic changes between cognitively healthy donors and AD patients, assessment of transcriptomic changes in individual cell types revealed slightly altered numbers of AD-associated DEGs (Figure 3). The top three most upregulated AD-associated genes (*STC1*, *BCYRN1*, *CBX3*) were shared between all cell types. A total of 70 AD-associated DEGs were common to all three cell types (Table S6). Statistical significance of the overlapping was confirmed through a multi-set intersection statistical test [42] and the lack of conflicting genes (same gene up and down-regulated in different cell types) was checked (Figure S6). The fibroblast/stromal-like cells are the most transcriptionally active with 91 up- and 136 down-regulated genes in AD (Figure 3a,b). Figure 3c shows the most up- and down-regulated differentially expressed genes in each cell type. For this, the GBC-like cells in different cell cycle phases were considered as one cell type. Interestingly, fibroblast/stromal- and myofibroblast-like cells exhibited the most unique, AD-associated DEGs (73 and 76 genes, respectively) (Figure 3b,c and Table S6). Highly up-regulated genes selectively in fibroblast/stromal- and myofibroblast-like cells included genes encoding for Brain Expressed X-Linked 1 (*BEX1*) and metallothionein 1X (*MT1X*), and Transcription Factor 4 (*TCF4*) and *RAB3B*, member RAS oncogene family (*RAB3B*), respectively. Unique to the AD GBC-like cells, for example, was the down-regulation of cellular communication network factor 2 (*CTGF* alias *CCN2*).

Using the 240 DEGs significantly up-regulated and down-regulated in AD we performed pathway analysis with Reactome database and GSVA and identified a total of 78 significantly enriched pathways (Figure 3d, Table S7). Enriched pathways were associated with RNA and protein metabolism, inflammatory processes, signal transduction, cell cycle, developmental biology, and the neuronal system (Figure 3d). Interestingly, our analysis revealed multiple pathways differentially expressed between the AD and control OM cells. For example, pathways related to seleno amino acid metabolism, selenocysteine synthesis, and viral and non-viral RNA processing were reduced in all AD OM cell types compared to the OM cells derived from cognitively healthy controls. However, transcriptional regulation by RUNX family transcription factors RUNX2 and RUNX3 were more enriched in AD OM cells.

Pathways enriched in GBC -like cells included several cell-cycle- and RHO GTPases related pathways, both of which were more enriched in AD when compared to control. On the other hand, pathways enriched in fibroblast/stromal- and myofibroblast-like cells included ECM organization (*TGFB1*, *MMP1*, *LUM*, *ELN*, *SERPINE1*, *HTRA1*, *TNC*, *THBS1*, *DCN*, *MMP14*, *CTSK*, *COL6A1*, *MFAP2*, *CAPN2*, *COL8A1*, *COL6A3*, *TKT*, *CTSB*), Interleukin-4 and Interleukin-13 signaling (*TGFB1*, *CCND1*, *MMP1*, *CCL2*, *FOS*), and Collagen degradation (*MMP14*, *MMP1*, *CTSK*, *COL6A1*, *COL8A1*, *COL6A3*, *CTSB*) (Figure 3d, Table S7).

Interestingly, the scRNA-seq data revealed alterations in the expression of mitochondrial genes in AD OM cells. Comparison of the AD-associated DEGs per cell type to MitoCarta3.0 database [43] revealed 21 DEGs in fibroblast/stromal-like cells, 17 DEGs in GBC-like cells, and 24 DEGs in myofibroblast-like cells (Table S8). Seven (*MT-ND2*, *MT-ND3*, *MT-ND4L*, *MT-ND6*, *NDUFA13*, *COX16*, *MT-ATP8*) in fibroblast/stromal-, four (*MT-ND2*, *MT-ND3*, *MT-ATP8*, *ATP5D*) in myofibroblast-, and six (*MT-ND2*, *MT-ND3*, *MT-ND4L*, *NDUFA13*, *COX16*, *MT-ATP8*) in GBC-like cells encode for subunits of the mitochondrial electron transport chain complexes.

**Figure 4:**
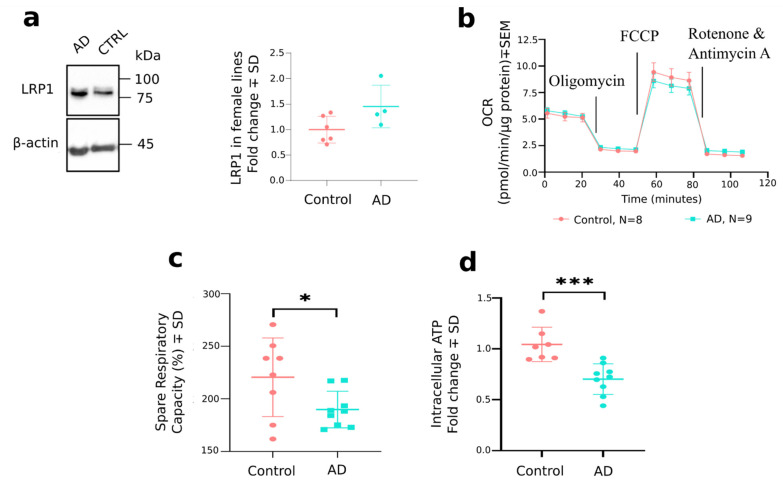
Levels of LRP1 and assays of mitochondrial function in OM cells derived from cognitively healthy individuals and patients with AD. (**a**) Western blot for LRP1 protein. Quantification from two blots normalized to β-actin. Both gels/blots were processed in parallel and cropped images are shown. *n* = 6 for cognitively healthy females and *n* = 4 for female patients with AD. Statistical testing between the control and AD cells was performed with t test (unpaired, two-tailed, *p* = 0.0644). (**b**,**c**) Mitochondrial respiration rates were assessed in OM cells derived from controls and patients with AD with the Seahorse MitoStress test (Agilent). *n* = 8 for cognitively healthy individuals and *n* = 9 for patients with AD. (**c**) Statistical testing for difference in spare respiratory capacity (%) between control and AD cells was performed with t test (unpaired, two-tailed). * *p* < 0.05. For the OCR graph, the data are presented as mean ± SEM and for the spare respiratory capacity (%) calculation as mean ± SD. (**d**) Intracellular levels of the ATP were measured from the OM cell cultures with a luminescence-based assay. *n* = 7 for cognitively healthy individuals and *n* = 9 for patients with AD. Statistical testing between the control and AD cells was performed with t test (unpaired, two-tailed) *** *p* ≤ 0.001.

*LRP1* encoding for LDL Receptor Related Protein 1 was found to be differentially expressed only between the AD and control OM fibroblast/stromal-like cells, being significantly up-regulated in AD. LRP1 contributes to the clearance of Aβ from the brain across the blood-brain barrier to the periphery (for review see [44]). Furthermore, in the brain endothelial cells LRP1 expression levels are reduced in AD, resulting in higher Aβ levels in the brain (for review see [44]). Since we observed increased secretion of Aβ_1–42_ from AD OM cells, we were interested in further studying the protein levels of LRP1 in OM cell cultures by Western blot. As a result, we observed the levels of LRP1 to be nearly significantly elevated in AD OM cells derived from female donors (0.4543 ± 0.2119, *p* = 0.0644). Interestingly, when male lines were also considered in the quantification, the slight difference in protein expression of LRP1 between AD and control OM cell cultures was lost (0.1458 ± 0.1463, *p* = 0.3338) (Figure 4a and Figure S4).

To validate the finding of differential expression of mitochondrial genes in AD OM cells, we measured the mitochondrial respiration rates of OM cells with the Seahorse MitoStress test (Agilent) (Figure 4b,c). The mitochondrial assay revealed a significant reduction in the spare respiratory capacity (%) of AD OM cells in comparison to cells derived from cognitively healthy individuals (−30.73 ± 13.87%, *p* =0.0425). Furthermore, we observed the intracellular levels of ATP to be significantly reduced in AD OM cell cultures compared to the control OM cells (−0.3413 ± 0.08004, *p* = 0.0008, Figure 4d) when assessed with a luminescence-based method.

## 4. Discussion

Olfactory impairment is an early symptom of AD, yet to date, the OM of AD patients remains poorly studied. Although previous reports demonstrated by histological assessment the presence of pathology typical for the disease, cell-type-specific changes and the cell functional alterations in the OM have remained under-explored. Our results demonstrate the increased secretion of Aβ_1–42_ and the increased ratio of secreted Aβ_1–42_/Aβ_1–40_ by OM cells derived from AD patients. Single-cell transcriptomic signatures of these cells revealed 240 differentially expressed AD-associated genes that are primarily involved in pathways related to RNA and protein metabolism, inflammatory processes, and signal transduction. Furthermore, the scRNA-sequencing proposed alterations in gene expression of mitochondrially located genes in AD OM cells, which were verified by functional assays, demonstrating altered mitochondrial respiration and a reduction of ATP production.

Our findings reveal that the OM cells can produce and secrete Aβ_1–40_ and Aβ_1–42_, with AD cells secreting significantly more Aβ_1–42_ than cognitively healthy control cells. This is in line with prior literature demonstrating that the nasal mucosa or nasal secretions of AD patients also exhibit increased levels of Aβ [7,8,9,10]. Furthermore, the amount of Aβ_1–42_ in the nasal area of transgenic Tg2576 mice modeling AD positively correlates with Aβ_1–42_ deposition in their brain [45]. The fact that AD OM cells secrete increased levels of Aβ demonstrates that the cells display the expected disease-specific pathological alterations, also observed in the AD brain. Skin fibroblasts derived from patients bearing a Swedish mutation of the amyloid precursor protein (APP KM670/671NL) have been shown to secrete significantly more beta-amyloid compared to the control fibroblasts [46]. We also have a population of cells classified as fibroblast/stromal cells in the OM cell cultures. One could thus think that based on the existing literature, the fibroblast/stromal cells of the OM cultures could contribute to this observed difference in secreted levels of Aβ_1–42_ between OM cell cultures derived from patients with AD and their controls. Supporting this hypothesis, (i) LRP1 is known to contribute to the clearance of beta-amyloid from the brain to the periphery (for review see [44]), (ii) we detected the levels of *LRP1* to be significantly increased selectively in the AD OM fibroblast/stromal-like cells, and (iii) we did not observe altered levels of the beta-amyloid influx receptor *RAGE* in AD OM fibroblast/stromal like cells (for review on the role of LRP1 in beta-amyloid clearance see [44]). To deduce the other possible reasons responsible for increased Aβ_1–42_ secretion in the AD OM cells, we found the expression of *MMP14* to be up-regulated in fibroblast/stromal- and GBC-like cells. Furthermore, *MMP14* was significantly upregulated in myofibroblast-like cells of AD patients. MMP14 is known to be involved in proteolytic cleavage of the LRP1 ectodomain (ligand binding α-chain) from the cell membrane, resulting in soluble LRP1 (for review see [44]). In this study, we detected a slight increase in the levels of LRP1 membrane-bound β-chain (85 kDa protein after proteolytic cleavage) in OM cells of female AD patients only. The Western blot results of LRP1 did not quite reach statistical significance, and there are at least a few possible reasons why the sequencing results were not reproduced at the protein level. First of all, the OM cell cultures used for extracting the protein samples for Western blot were not purified for the fibroblast/stromal-like cells, the cell type in which the statistically significant difference was observed in female lines at the mRNA level. Rather, the protein sample consists of a mix of different cell types, in which cell-type-specific changes can be lost. Secondly, we are limited by the small cohort of female cell lines available for protein analyses. Levels of LRP1 have been quantified in human AD brains previously by others, and temporal and regional changes in LRP1 expression are observed (for review see [47]). Further studies on the role of LRP1 in AD, and potentially also its gender- and tissue-specific changes, are thus needed.

Beyond the OM, olfactory sensory cues are processed by the olfactory bulb, olfactory tract, piriform cortex, amygdala, entorhinal cortex, and hippocampus [1]. ScRNA-seq results by Grubman et al. on the AD entorhinal cortex and our findings have the following eight common DEGs between the control and AD groups [15]: *SERPINE1*, *IFI27*, *MT-ND2*, *MT-ND3*, *MAP1B*, *BCYRN1*, *FTH1,* and *HES1*. The presence of these common DEGs highlights the importance of these genes in AD independently of the tissue type assessed and suggests that the entorhinal cortex and OM, both vulnerable to early AD pathogenesis, display disease-specific alterations that may have the potential for targeting by therapeutic approaches. The other highly differentially expressed genes mentioned have also been reported to show perturbations in the brain, and we consider them interesting given that they are also altered in the AD OM cells. To mention some, the mitochondrial ETC complex V gene *MT-ATP8* encoding for a subunit of the ATP synthase was up-regulated in all cell types of the cultured AD OM (for all cell-types log2FC > 0.84). Notably, an increase in the mRNA expression of *MT-ATP8* has been shown previously in the frontal cortex of AD-affected brains [48]. Furthermore, protein levels of the matrix metalloproteinase 1, encoded by *MMP1*, have been shown to be elevated in the cortex of individuals affected by AD [49], which is in line with our observations for increased mRNA levels of *MMP1* in AD OM. Our results indicated that the gene expression level of *MT2A* (metallothionein 2A) was significantly up-regulated in all AD OM-derived cells, being the most upregulated in fibroblast/stromal-like cells, while no changes in *MT3* expression were observed. Previous studies have also demonstrated an increase in *MT2A* expression in skin fibroblasts of AD patients [50], supporting our observation. Interestingly, the gene encoding for metallothionein 1X (*MT1X*) was significantly up-regulated solely in fibroblast/stromal-like cells (log2FC = 0.45) derived from AD OM. The expression of metallothionein proteins is suggested to become elevated in response to increased amounts of metals or reactive oxygen species (ROS) in the cell (for review see [51]). On the other hand, when *MT2A* is overexpressed in HEK cells, the cells display reduced capacity for oxidative phosphorylation, thereby affecting their mitochondrial function [52]. These findings suggest that alterations in mitochondrial function may occur in AD OM cells in a similar manner as observed in other cells in AD (reviewed in [53,54]). This idea is supported by the finding that both fibroblast/stromal- and GBC-like cells derived from the AD OM displayed up-regulation of several genes encoding for complex I subunits (*MT-ND2*, *MT-ND3*, *NDUFA13*, *MT-ND4L*). Interestingly, several mitochondrial-encoded *OXPHOS* genes have been shown to be upregulated in circulating white blood cells of patients with AD and individuals with mild cognitive impairment [55]. Furthermore, *MT-ND6* was up-regulated selectively in the fibroblast/stromal-like cells. Indicative of potential mitochondrial dysfunction, our results revealed significantly reduced spare respiratory capacity when the oxygen consumption rate of the AD OM cells was measured. The spare respiratory capacity (%) of the cells is calculated by dividing the maximal respiration values (OCR values after FCCP injection) with values of the cell’s basal respiration (OCR values at the beginning of the assay). This observation is in line with published reports on the differences observed in skin fibroblasts derived from patients with AD [56]. Furthermore, the levels of ATP were significantly reduced in AD OM cells, also indicative of mitochondrial dysfunction. The reduction in functional measures of the AD OM cell mitochondria by both spare respiratory capacity and intracellular ATP levels may indicate the reduced ability of the AD OM cell mitochondria to combat stress or cope with increased energy demands. Detailed studies into the connections between heavy metal-binding proteins or oxidative stress and mitochondrial function in OM would form an interesting future direction of research.

Multiple genome-wide association studies have found pathways related to metabolism and immune system alteration in AD [57,58,59], thus supporting our findings of altered DEGs related to pathways including RNA and protein metabolism, inflammatory processes, and signal transduction. We observed that immune system pathways were significantly enriched in the AD OM cells, which also aligns with earlier published studies [60,61]. In addition, inflammatory processes are a well-described pathological feature of AD (review [62]).

Furthermore, we report pathways related to seleno amino acids to be reduced in all cell types of the AD OM. As one example, the gene encoding for selenoprotein, *GPX4*, was significantly down-regulated in all identified cell types of the cultured AD OM cells. The GPX4 protein can inhibit ferroptosis, a phenomenon that has been reported to be related to neurodegenerative diseases (review [63]).

The OM, located at the rooftop of the nasal cavity, is exposed to the inhaled air in living individuals, and thus the cells of the OM are in direct contact with airborne agents such as viruses and other pathogens. Considering this, the alterations we observed in the viral and non-viral RNA processing pathways of OM cells can be expected. Similar to our study, pathways related to viral RNA processing have been recently shown to be significantly altered also in RNA samples derived from peripheral blood of patients with LOAD [64].

In the adult brain, new neurons form only in certain anatomical locations; in the dentate gyrus of the hippocampus (particularly in the subgranular zone) and in the subventricular zone (SVZ). Interestingly the newly formed neurons in the SVZ eventually migrate to the olfactory bulb for maturation and differentiation (review [65]. As the OM is (i) a tissue with life-long regenerative capacity (review [1], (ii) in contact with olfactory bulb [review [1], and (iii) adult neurogenesis is reported to be significantly reduced in AD affected human brains [66], it is conceivable that the observed reduction in the enrichment of pathways associated with RNA and protein metabolism, developmental biology and the neuronal system are related to processes occurring in OM cells derived from individuals suffering from age-related diseases such as AD.

Furthermore, one would expect enrichment of pathways related to ECM organization and collagen degradation in the fibroblast/stromal- and myofibroblast-like cells due to the nature of the OM cells (review [67]). Pathways for transcriptional regulation by RUNX2 and RUNX3 were more enriched in all OM cell types derived from donors with AD. Interestingly, the overexpression of the *RUNX2* gene, encoding for transcription factor RUNX2, in cell lines is known to alter gene expression of several mitochondrially encoded genes and also the transcripts for *LRP1* [68]. Two of the mitochondrially encoded genes shown with altered gene expression after *RUNX2* overexpression (*MT-ND4L*, *MT-ND6*) were also found to be significantly differentially expressed between the AD and control OM fibroblast/stromal-like cells. Based on the above-mentioned observations regarding the enriched pathways, both by OM cell type and AD OM over control, we believe that our data shed new light on the disease-related cellular and molecular events occurring in the OM, which has thus far been little studied.

Clusterin (encoded by *CLU*), also known as apolipoprotein J, is strongly linked to AD and up-regulated in patient brains as a protective response (for review see [69]). Interestingly, in our study, *CLU* was down-regulated in AD OM-derived fibroblast/stromal-like cells and GBC-like cells. Prior studies have shown that in lung fibroblasts, *CLU* expression is reduced by transforming growth factor β 1 (TGFβ1) [70], which is in contrast with neurons and astrocytes, where TGFβ induces clusterin expression (for review see [69]). It is, therefore, possible that clusterin alterations in AD are cell-type specific and related to TGFβ levels. Furthermore, it is known that the fibroblast proliferation promoting effects of serum are diminished in cells expressing low levels of *CLU* [70]. This, together with our results of reduced numbers of fibroblast/stromal cells in AD OM cultures grown in serum-containing media, may suggest the involvement of clusterin in the proliferation of OM-derived fibroblast/stromal cells in vitro.

We identified fibroblast/stromal-like cells, myofibroblast-like cells, and GBC-like cells as the cell types dominantly present in the cultures of OM of both cognitively healthy controls and AD patients. It is likely that the cultures do not contain all the cell types present in the OM in vivo. This is to be expected given that the more robust cell types such as fibroblast/stromal-like cells tend to survive the culturing conditions better and dominate the more sensitive and low abundance cells such as sensory neurons. Furthermore, the culture conditions, including culture media composition, are likely to better promote the growth of some cell types while lacking support for others. To mention one, Newman et al. have shown that neurons do not survive the primary cultures of OM cells derived from adult mice, but they can be induced by the right cocktail of growth factors [71]. We believe that the observed similarities in enriched pathways between the cell clusters identified as GBCs in the current study and in the previously published GBCs in vivo by Durante et al. [26] emphasize that the observed AD-related alterations in gene expression are not a consequence of the culturing conditions to which the cells are subjected. The lack of neurons in the cultured cells is most likely also the reason for the absence of a tau alteration in the AD OM. Although hyperphosphorylated tau was detected in neurites in AD OM histological sections [9], we did not observe a change in levels of total or phosphorylated tau between AD and control OM cells. This lack of change is not surprising given that mature neurons were not detected in our OM cell cultures, where tau is expressed endogenously [72]. Furthermore, we consider the key AD-related changes of the OM cells to be preserved by passaging of the cells since the gene expression alterations identified in the sequencing of cells at passages 2–3 were supported by the outcome of the functional assays related to the mitochondrial function of the OM cells at the passages 4–6. We propose this culture model of OM cells to be useful for identifying disease-related alterations in these cells, and furthermore, for future use in drug screening purposes or testing of new therapeutic molecules

The results shown here demonstrate that OM cells of AD patients display disease-associated alterations linked to increased secretion of Aβ and altered energy metabolism, as well as 240 differentially expressed AD-associated genes that are primarily involved in pathways related to RNA and protein metabolism, inflammatory processes, and signal transduction. The OM cells present high research potential in complex age-related diseases including late-onset AD.

## Data Availability

The data presented in this study are available on request from the corresponding author. Single cell RNA sequencing data are available from the European Genome-phenome Archive (EGA, https://ega-archive.org/, 10 February 2022) under the accession ID EGAS00001006019.

## Supplementary Materials

The following supporting information can be downloaded at: https://www.mdpi.com/article/10.3390/cells11040676/s1, Figure S1: The levels of secreted Aβ1–42 were not found to correlate to the status of the donor’s sense of smell or APOE genotype; Figure S2: Quality control results of the scRNA-seq data; Figure S3: Cell cycle scores based on the expression of G2/M and S phase markers in each cell; Figure S4: Uncropped immunoblot images for quantification of LRP1; Figure S5: Statistical analysis of the overlapping pathways for GBC-like cells; Figure S6: Statistical analysis of the overlapping DE genes by cell type; Figure S7: The heatmap representing a Pearson correlation matrix created using the marker genes used to identify the different cell types; Figure S8: Feature plots for a subset of the 30 most up-regulated and significant differentially expressed genes between the clusters used for defining the cell populations; Figure S9: Feature plots (a) and heatmap (b) displaying cell-type specific alterations for a subset of DEGs showing transcriptional alterations between AD and control OM cells; Table S1: The top 30 most up-regulated and significant differentially expressed genes between the clusters; Table S2: Cell annotation; Table S3: Number of cells per each cell type for each individual; Table S4: 76 common pathways between GBC cells derived from cultured olfactory mucosa cells in this experiment and in GBCs derived from the olfactory mucosa biopsy (Durante et al. Nat Neurosci 2020, *23*, 323–326) and their related functions in the cell; Table S5: List of AD-associated differentially expressed genes between cognitively healthy individuals and patients with AD; Table S6: List of genes detected as differentially expressed between AD and controls for each cell type and linked to Alzheimer’s Disease in literature; Table S7: List of significantly enriched pathways found both with Gene Set Variation Analysis (GSVA) and Reactome database for differentially expressed AD-associated genes (FDR < 0.1); Table S8: Comparison of the AD-associated DEGs per cell type to the MitoCarta3.0, inventory of the mammalian mitochondrial proteome, dataset (Rath et al. Nucleic Acids Res. 2021, *49*, 1541–1547).

## Appendix A

### ScRNA-Seq, Additional Details of the Analysis

Quality Control and Downstream Analysis: Working separately on the controls and AD samples data Matrixes, hashtag oligos assays were normalized with NormalizeData() function applying the centered log-ratio (CLR) transformation. Seurat’s HTODemux was ran with default parameters on the hashtag-count matrices to demultiplex interested cell barcodes. HTO count distribution was checked and cells were classified into singlet/doublet/negative. With HTODemux single cells were assigned back to their samples of origin. After the demultiplexing step, doublets were removed using the subset() function and the cell filtering was performed separately for the three libraries to remove the low-quality cells. For AD, the samples were prepared in two libraries. In library 1, we kept only cells with nFeature > 750 and nFeature < 6000, nCount_RNA < 40000 and mitochondrial content < 15%, and for library 2 the settings were nFeature > 750 and nFeature < 7000, nCount_RNA < 55000 and mitochondrial content < 12%. In Control samples, we retained only cells with nFeature > 750 and nFeature < 7000, nCount_RNA < 50000 and mitochondrial content < 15%. Control and AD samples were integrated using Seurat standard integration pipeline. The function SplitObject() was used to separate, inside the three libraries, each sample which was—except for negative cells omitted in the integration process—individually normalized using the NormalizeData() function and the standard ‘LogNormalize’ method. From each of the eight samples, the 5000 most variable features were extracted using the FindVariableFeatures() function applying the ‘vst’ method. Thereafter, all the samples were integrated, first SelectIntegrationFeatures() function was used to select the best 5000 features for the integration, anchors between samples were extracted using the 5000 selected integration features and 30 dimensions as parameters in the FindIntegrationAnchors() function, then, all the eight samples were integrated using the IntegrateData() function. The resulting Seurat object was then scaled with ScaleData() function and PCA was applied using RunPCA() function. With the FindNeighbors() function, using the first 30 dimensions, the Shared Nearest Neighbor Graph was constructed and cells clustered using the FindClusters() function. Different clustering resolutions were tested. The cluster resolution value 0.2 was the best to represent cell-type annotation. RunUMAP() function was used to apply UMAP dimensional reduction and obtain a spatial visualization of the cells. For each cluster the top 30 over-expressed genes were identified with FindAllMarkers() function using the Wilcoxon Rank Sum test and setting the min.pct and logfc.threshold parameters to 0.3. (Table S1). Gene-gene correlation of the top 30 over-expressed genes of each cell type was explored using the function rcorr() of the R package Hmisc (Figure S7). Feature plots for a subset of the 30 most up-regulated and significant differentially expressed genes between the clusters used for defining the cell populations and for a subset of DEGs displaying transcriptional alterations between the AD and control OM cells were produced using the FeaturePlot() function of Seurat package (Figures S8 and S9). The cell cycle phase score was calculated for each cell using the CellCycleScoring() function provided by the Seurat package.

Differential Expression and Enrichment: The Seurat FindMarkers() function and the Wilcoxon Rank Sum test were used to perform the differential expression analysis between all the cells of the Alzheimer’s disease and control libraries. The same comparison between control and AD libraries was also performed for each cell-type subpopulation. Seurat FindAllMarkers() function was used to retrieve representative markers for GBC clusters. Next, the enrichment analysis was performed to check the involvement of GBC cells, belonging to these two distinct datasets, in similar biological processes.

Finally, a multi-set intersection statistical test [42] was applied to prove that the sharing of common GBC pathways between datasets was not due to chance, and also to explore the significance of the overlapping differentially expressed genes between the different cell types.

## References

[1] Marin C., Vilas D., Langdon C., Alobid I., López-Chacón M., Haehner A., Hummel T., Mullol J. (2018). Olfactory Dysfunction in Neurodegenerative Diseases. Curr. Allergy Asthma Rep..

[2] Féron F., Perry C., McGrath J.J., Mackay-Sim A. (1998). New techniques for biopsy and culture of human olfactory epithelial neurons. Arch. Otolaryngol. Head Neck Surg..

[3] Jung H.J., Shin I.S., Lee J.E. (2019). Olfactory function in mild cognitive impairment and Alzheimer’s disease: A meta-analysis. Laryngoscope.

[4] Sohrabi H.R., Bates K.A., Rodrigues M., Taddei K., Laws S.M., Lautenschlager N.T., Dhaliwal S.S., Johnston A.N.B., Mackay-Sim A., Gandy S. (2009). Olfactory dysfunction is associated with subjective memory complaints in community-dwelling elderly individuals. J. Alzheimers Dis..

[5] Sohrabi H.R., Bates K.A., Weinborn M.G., Johnston A.N.B., Bahramian A., Taddei K., Laws S.M., Rodrigues M., Morici M., Howard M. (2012). Olfactory discrimination predicts cognitive decline among community-dwelling older adults. Transl. Psychiatry.

[6] Yoo S.J., Son G., Bae J., Kim S.Y., Yoo Y.K., Park D., Baek S.Y., Chang K.-A., Suh Y.-H., Lee Y.-B. (2020). Longitudinal profiling of oligomeric Aβ in human nasal discharge reflecting cognitive decline in probable Alzheimer’s disease. Sci. Rep..

[7] Liu Z., Kameshima N., Nanjo T., Shiino A., Kato T., Shimizu S., Shimizu T., Tanaka S., Miura K., Tooyama I. (2018). Development of a High-Sensitivity Method for the Measurement of Human Nasal Aβ 42, Tau, and Phosphorylated Tau. J. Alzheimers Dis..

[8] Kim Y.H., Lee S.M., Cho S., Kang J.-H., Minn J.-K., Park H., Choi S.H. (2019). Amyloid beta in nasal secretions may be a potential biomarker of Alzheimer’s disease. Sci. Rep..

[9] Arnold S.E., Lee E.B., Moberg P.J., Stutzbach L., Kazi H., Han L.-Y., Lee V.M.Y., Trojanowski J.Q. (2010). Olfactory epithelium amyloid-β and paired helical filament-tau pathology in Alzheimer disease. Ann. Neurol..

[10] Ayala-Grosso C.A., Pieruzzini R., Diaz-Solano D., Wittig O., Abrante L., Vargas L., Cardier J. (2015). Amyloid-Aβ peptide in olfactory mucosa and mesenchymal stromal cells of mild cognitive impairment and Alzheimer’s disease patients. Brain Pathol..

[11] Talamo B.R., Rudel R.A., Kosik K.S., Lee V.M.-Y., Neff S., Adelman L., Kauer J.S. (1989). Pathological changes in olfactory neurons in patients with Alzheimer’s disease. Nature.

[12] Lee J.H., Goedert M., Hill W.D., Lee V.M.Y., Trojanowski J.Q. (1993). Tau Proteins Are Abnormally Expressed in Olfactory Epithelium of Alzheimer Patients and Developmentally Regulated in Human Fetal Spinal Cord. Exp. Neurol..

[13] Ghanbari H.A., Ghanbari K., Harris P.L.R., Jones P.K., Kubat Z., Castellani R.J., Wolozin R.L., Smith M.A., Perry G. (2004). Oxidative damage in cultured human olfactory neurons from Alzheimer’s disease patients. Aging Cell.

[14] Wolozin B., Lesch P., Lebovics R., Sunderland T. (1993). Olfactory neuroblasts from Alzheimer donors: Studies on APP processing and cell regulation. Biol. Psychiatry.

[15] Grubman A., Chew G., Ouyang J.F., Sun G., Choo X.Y., MacLean C., Simmons R.K., Buckberry S., Vargas-Landin D.B., Poppe D. (2019). A single-cell atlas of entorhinal cortex from individuals with Alzheimer’s disease reveals cell-type-specific gene expression regulation. Nat. Neurosci..

[16] Mathys H., Davila-Velderrain J., Peng Z., Gao F., Mohammadi S., Young J.Z., Menon M., He L., Abdurrob F., Jiang X. (2019). Single-cell transcriptomic analysis of Alzheimer’s disease. Nature.

[17] Olah M., Menon V., Habib N., Taga M.F., Ma Y., Yung C.J., Cimpean M., Khairallah A., Coronas-Samano G., Sankowski R. (2020). Single cell RNA sequencing of human microglia uncovers a subset associated with Alzheimer’s disease. Nat. Commun..

[18] Zhou Y., Song W.M., Andhey P.S., Swain A., Levy T., Miller K.R., Poliani P.L., Cominelli M., Grover S., Gilfillan S. (2020). Human and mouse single-nucleus transcriptomics reveal TREM2-dependent and TREM2-independent cellular responses in Alzheimer’s disease. Nat. Med..

[19] Kashima Y., Sakamoto Y., Kaneko K., Seki M., Suzuki Y., Suzuki A. (2020). Single-cell sequencing techniques from individual to multiomics analyses. Exp. Mol. Med..

[20] Aldridge S., Teichmann S.A. (2020). Single cell transcriptomics comes of age. Nat. Commun..

[21] Martinez-Jimenez C.P., Eling N., Chen H.C., Vallejos C.A., Kolodziejczyk A.A., Connor F., Stojic L., Rayner T.F., Stubbington M.J.T., Teichmann S.A. (2017). Aging increases cell-to-cell transcriptional variability upon immune stimulation. Science.

[22] Kumar M.P., Du J., Lagoudas G., Jiao Y., Sawyer A., Drummon D.C., Lauffenburger D.A., Raue A. (2018). Analysis of Single-Cell RNA-Seq Identifies Cell-Cell Communication Associated with Tumor Characteristics. Cell Rep..

[23] Vistain L.F., Tay S. (2021). Single-Cell Proteomics. Trends Biochem. Sci..

[24] Yang L., George J., Wang J. (2020). Deep Profiling of Cellular Heterogeneity by Emerging Single-Cell Proteomic Technologies. Proteomics.

[25] Rubakhin S.S., Lanni E.J., Sweedler J.V. (2013). Progress toward single cell metabolomics. Curr. Opin. Biotechnol..

[26] Durante M.A., Kurtenbach S., Sargi Z.B., Harbour J.W., Choi R., Kurtenbach S., Goss G.M., Matsunami H., Goldstein B.J. (2020). Single-cell analysis of olfactory neurogenesis and differentiation in adult humans. Nat. Neurosci..

[27] Oliva A.D., Gupta R., Issa K., Hachem R.A., Jang D.W., Wellford S.A., Moseman A.E., Matsunami H., Goldstein B.J. (2022). Aging-related olfactory loss is associated with olfactory stem cell transcriptional alterations in humans. J. Clin. Investig..

[28] Jack C.R., Bennett D.A., Blennow K., Carrillo M.C., Dunn B., Haeberlein S.B., Holtzman D.M., Jagust W., Jessen F., Karlawish J. (2018). NIA-AA Research Framework: Toward a biological definition of Alzheimer’s disease. Alzheimers Dement..

[29] Morris J.C., Heyman A., Mohs R.C., Hughes J.P., van Belle G., Fillenbaum G., Mellits E.D., Clark C. (1989). The consortium to establish a registry for Alzheimer’s disease (CERAD). Part I. Clinical and neuropsychological assessment of Alzheimer’s disease. Neurology.

[30] Mirra S.S., Heyman A., McKeel D., Sumi S.M., Crain B.J., Brownlee L.M., Vogel F.S., Hughes J.P., van Belle G., Berg L. (1991). The consortium to establish a registry for Alzheimer’s disease (CERAD). Part II. Standardization of the neuropathologic assessment of Alzheimer’s disease. Neurology.

[31] Huovinen J., Kastinen S., Komulainen S., Oinas M., Avellan C., Frantzen J., Rinne J., Ronkainen A., Kauppinen M., Lönnrot K. (2016). Familial idiopathic normal pressure hydrocephalus. J. Neurol Sci..

[32] Hummel T., Konnerth C.G., Rosenheim K., Kobal G. (2001). Screening of olfactory function with a four-minute odor identification test: Reliability, normative data, and investigations in patients with olfactory loss. Ann. Otol. Rhinol. Laryngol..

[33] Murrell W., Féron F., Wetzig A., Cameron N., Splatt K., Bellette B., Blanco J., Perry C., Lee G., Mackay-Sim A. (2005). Multipotent stem cells from adult olfactory mucosa. Dev. Dyn..

[34] Hao Y., Hao S., Andersen-Nissen E., Mauck W.M., Zheng S., Butler A., Lee M.J., Wilk A.J., Darby C., Zager M. (2021). Integrated analysis of multimodal single-cell data. Cell.

[35] Chen E.Y., Tan C.M., Kou Y., Duan Q., Wang Z., Vaz Meirelles G., Clark N.R., Ma’ayan A. (2013). Enrichr: Interactive and Collaborative HTML5 Gene List Enrichment Analysis Tool. BMC Bioinform..

[36] Kuleshov M.V., Jones M.R., Rouillard A.D., Fernandez N.F., Duan Q., Wang Z., Koplev S., Jenkins S.L., Jagodnik K.M., Lachmann A. (2016). Enrichr: A comprehensive gene set enrichment analysis web server 2016 update. Nucleic Acids Res..

[37] Xie Z., Bailey A., Kuleshov M.V., Clarke D.J.B., Evangelista J.E., Jenkins S.J., Lachmann A., Wojciechowicz M.L., Kropiwnicki E., Jagodnik K.M. (2021). Gene Set Knowledge Discovery with Enrichr. Curr. Protoc..

[38] Greene C.S., Krishnan A., Wong A.K., Ricciotti E., Zelaya R.A., Himmelstein D.S., Zhang R., Hartmann B.M., Zaslavsky E., Sealfon S.C. (2015). Understanding multicellular function and disease with human tissue-specific networks. Nat. Genet..

[39] Hänzelmann S., Castelo R., Guinney J. (2013). GSVA: Gene Set Variation Analysis for Microarray and RNA-Seq Data. BMC Bioinform..

[40] Jassal B., Matthews L., Viteri G., Gong C., Lorente P., Fabregat A., Sidiropoulos K., Cook J., Gillespie M., Haw R. (2020). The reactome pathway knowledgebase. Nucleic Acids Res..

[41] Fabregat A., Sidiropoulos K., Viteri G., Marin-Garcia P., Ping P., Stein L., D’Eustachio P., Hermjakob H. (2018). Reactome diagram viewer: Data structures and strategies to boost performance. Bioinformatics.

[42] Wang M., Zhao Y., Zhang B. (2015). Efficient test and visualization of multi-set intersections. Sci. Rep..

[43] Rath S., Sharma R., Gupta R., Ast T., Chan C., Durham T.J., Goodman R.P., Grabarek Z., Haas M.E., Hung W.H.W. (2021). MitoCarta3.0: An updated mitochondrial proteome now with sub-organelle localization and pathway annotations. Nucleic Acids Res..

[44] Ramanathan A., Nelson A.R., Sagare A.P., Zlokovic B.V. (2015). Impaired vascular-mediated clearance of brain amyloid beta in Alzheimer’s disease: The role, regulation and restoration of LRP1. Front. Aging Neurosci..

[45] Kameshima N., Nanjou T., Fukuhara T., Yanagisawa D., Tooyama I. (2012). Correlation of Aβ deposition in the nasal cavity with the formation of senile plaques in the brain of a transgenic mouse model of Alzheimer’s disease. Neurosci. Lett..

[46] Johnston J.A., Cowburn R.F., Norgren S., Wiehager B., Venizelos N., Winblad B., Vigo-Pelfrey C., Schenk D., Lannfelt L., O’Neill C. (1994). Increased beta-amyloid release and levels of amyloid precursor protein (APP) in fibroblast cell lines from family members with the Swedish Alzheimer’s disease APP670/671 mutation. FEBS Lett..

[47] Shinohara M., Tachibana M., Kanekiyo T., Bu G. (2017). Role of LRP1 in the pathogenesis of Alzheimer’s disease: Evidence from clinical and preclinical studies. J. Lipid Res..

[48] Manczak M., Park P.S., Jung Y., Reddy P.H. (2004). Differential expression of oxidative phosphorylation genes in patients with Alzheimer’s disease: Implications for early mitochondrial dysfunction and oxidative damage. Neuromolecular. Med..

[49] Leake A., Morris C.M., Whateley J. (2000). Brain matrix metalloproteinase 1 levels are elevated in Alzheimer’s disease. Neurosci. Lett..

[50] Lanni C., Nardinocchi L., Puca R., Stanga S., Uberti D., Memo M., Govoni S., D’Orazi G., Racchi M. (2010). Homeodomain Interacting Protein Kinase 2: A Target for Alzheimer’s Beta Amyloid Leading to Misfolded p53 and Inappropriate Cell Survival. PLoS ONE.

[51] Lindeque J.Z., Levanets O., Louw R., van der Westhuizen F.H. (2010). The Involvement of Metallothioneins in Mitochondrial Function and Disease. Curr. Protein Pept. Sci..

[52] Bragina O., Gurjanova K., Krishtal J., Kulp M., Karro N., Tõugu V., Palumaa P. (2015). Metallothionein 2A affects the cell respiration by suppressing the expression of mitochondrial protein cytochrome c oxidase subunit II. J. Bioenerg Biomembr..

[53] Chakravorty A., Jetto C.T., Manjithaya R. (2019). Dysfunctional Mitochondria and Mitophagy as Drivers of Alzheimer’s Disease Pathogenesis. Front. Aging Neurosci..

[54] Wang W., Zhao F., Ma X., Perry G., Zhu X. (2020). Mitochondria dysfunction in the pathogenesis of Alzheimer’s disease: Recent advances. Mol. Neurodegener..

[55] Lunnon K., Keohane A., Pidsley R., Newhouse S., Riddoch-Contreras J., Thubron E.B., Devall M., Soininen H., Kłoszewska I., Mecocci P. (2017). Mitochondrial genes are altered in blood early in Alzheimer’s disease. Neurobiol. Aging.

[56] Bell S.M., de Marco M., Barnes K., Shaw P.J., Ferraiuolo L., Blackburn D.J., Mortiboys H., Venneri A. (2020). Deficits in mitochondrial spare respiratory capacity contribute to the neuropsychological changes of Alzheimer’s disease. J. Pers. Med..

[57] Lambert J.C., Grenier-Boley B., Chouraki V., Heath S., Zelenika D., Fievet N., Hannequin D., Pasquier F., Hanon O., Brice A. (2010). Implication of the immune system in Alzheimer’s disease: Evidence from genome-wide pathway analysis. J. Alzheimers Dis..

[58] Jones L., Holmans P.A., Hamshere M.L., Harold D., Moskvina V., Ivanov D., Pocklington A., Abraham R., Hollingworth P., Sims R. (2010). Genetic Evidence Implicates the Immune System and Cholesterol Metabolism in the Aetiology of Alzheimer’s Disease. PLoS ONE.

[59] Jiang Q., Jin S., Jiang Y., Liao M., Feng R., Zhang L., Liu G., Hao J. (2017). Alzheimer’s Disease Variants with the Genome-Wide Significance are Significantly Enriched in Immune Pathways and Active in Immune Cells. Mol. Neurobiol..

[60] Pillai J.A., Maxwell S., Bena J., Bekris L.M., Rao S.M., Change M., Lamb B.T., Leverenz L.B. (2019). Key inflammatory pathway activations in the MCI stage of Alzheimer’s disease. Ann. Clin. Transl. Neurol..

[61] Chen J., Xie C., Zhao Y., Li Z., Xu P., Yao L. (2016). Gene expression analysis reveals the dysregulation of immune and metabolic pathways in Alzheimer’s disease. Oncotarget.

[62] Leng F., Edison P. (2021). Neuroinflammation and microglial activation in Alzheimer disease: Where do we go from here?. Nat. Rev. Neurol..

[63] Abdalkader M., Lampinen R., Kanninen K.M., Malm T.M., Liddell J.R. (2018). Targeting Nrf2 to Suppress Ferroptosis and Mitochondrial Dysfunction in Neurodegeneration. Front. Neurosci..

[64] Fernández-Martínez J.L., Álvarez-Machancoses Ó., de Andrés-Galiana E.J., Bea G., Kloczkowski A. (2020). Robust Sampling of Defective Pathways in Alzheimer’s Disease. Implications in Drug Repositioning. Int. J. Mol. Sci..

[65] Ming G.-L., Song H. (2011). Adult neurogenesis in the mammalian brain: Significant answers and significant questions. Neuron.

[66] Moreno-Jiménez E.P., Flor-García M., Terreros-Roncal J., Rábano A., Cafini F., Pallas-Bazarra N., Ávila J., Llorens-Martín M. (2019). Adult hippocampal neurogenesis is abundant in neurologically healthy subjects and drops sharply in patients with Alzheimer’s disease. Nat. Med..

[67] Kalluri R. (2016). The biology and function of fibroblasts in cancer. Nat. Rev. Cancer.

[68] Kuhlwilm M., Davierwala A., Pääbo S. (2013). Identification of putative target genes of the transcription factor RUNX2. PLoS ONE.

[69] Nuutinen T., Suuronen T., Kauppinen A., Salminen A. (2009). Clusterin: A forgotten player in Alzheimer’s disease. Brain Res. Rev..

[70] Peix L., Evans I.C., Pearce D.R., Simpson J.K., Maher T.M., McAnulty R.J. (2018). Diverse functions of clusterin promote and protect against the development of pulmonary fibrosis. Sci. Rep..

[71] Newman M.P., Féron F., Mackay-Sim A. (2000). Growth factor regulation of neurogenesis in adult olfactory epithelium. Neuroscience.

[72] Kovacs G.G. (2020). Astroglia and Tau: New Perspectives. Front. Aging Neurosci..

