# Supplementary material for: Single-Cell RNA-Seq Analysis of Olfactory Mucosal Cells of Alzheimer’s Disease Patients
Source: Cells. 2022 Feb 15;11(4):676. doi: 10.3390/cells11040676 (PMC8870160; doi:10.3390/cells11040676)
Supplement: Supplementary file 1 [file cells-11-00676-s001.zip › Figures S1-S9.pdf]

Supplementary material

Supplementary Figures

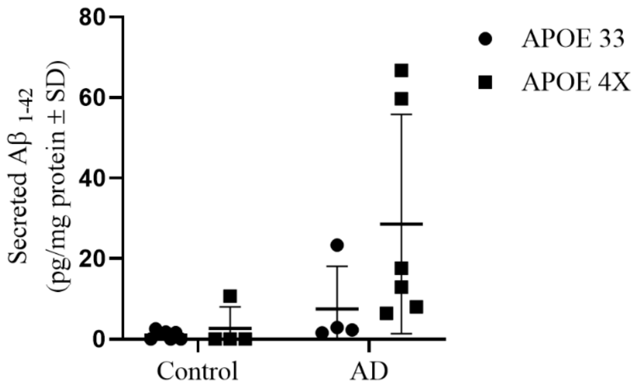

Figure S1. The levels of secreted Aβ$_{1-42}$ were not found to correlate to the donor's *APOE* genotype. OM cells harvested from biopsies were cultured for 7 days prior ELISA assay, assessing levels of Aβ$_{1-42}$ in media collected from OM cells. The results were normalized to the total amount of protein measured from cell lysates and then separated to subgroups based on the donor's *APOE* genotype. N= 11 donors in total for controls and 10 for AD. Quantification of secreted Aβ$_{1-42}$ between control and AD subgroups was performed with two-way ANOVA. A statistically significant difference was detected between the control and AD in the amount of secreted Aβ$_{1-42}$ (P = 0.0338), but not between the *APOE* genotypes. Data is presented as mean values ± SD.

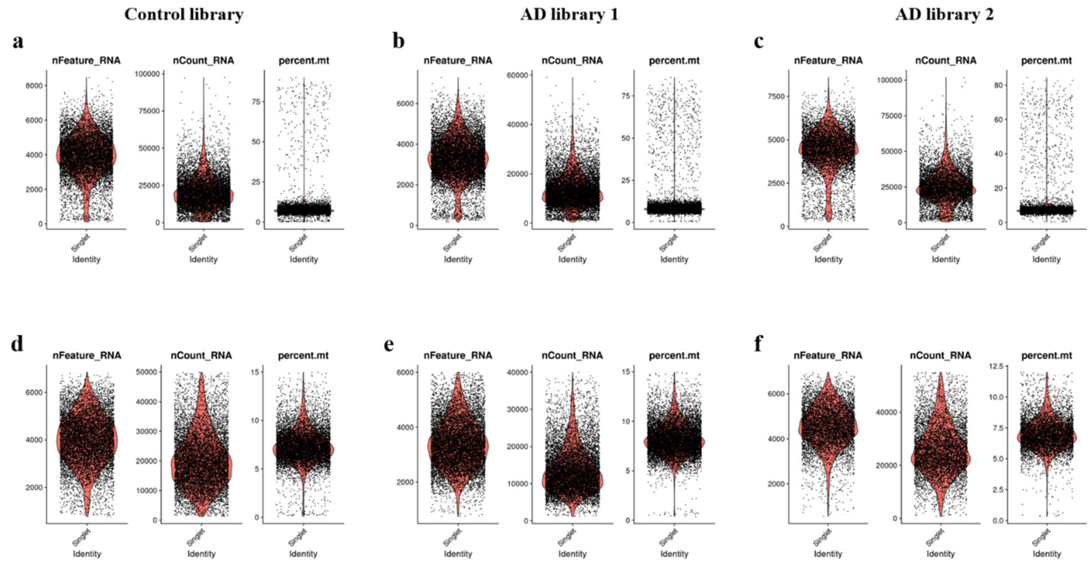

Figure S2. Quality control results of the scRNA-seq data. Results before filtering for (**A**) control library and for (**B**) AD library 1, (**C**) AD library 2, and in addition after filtering (**D**) for control library, (**E**) AD library 1, and (**F**) AD library 2.

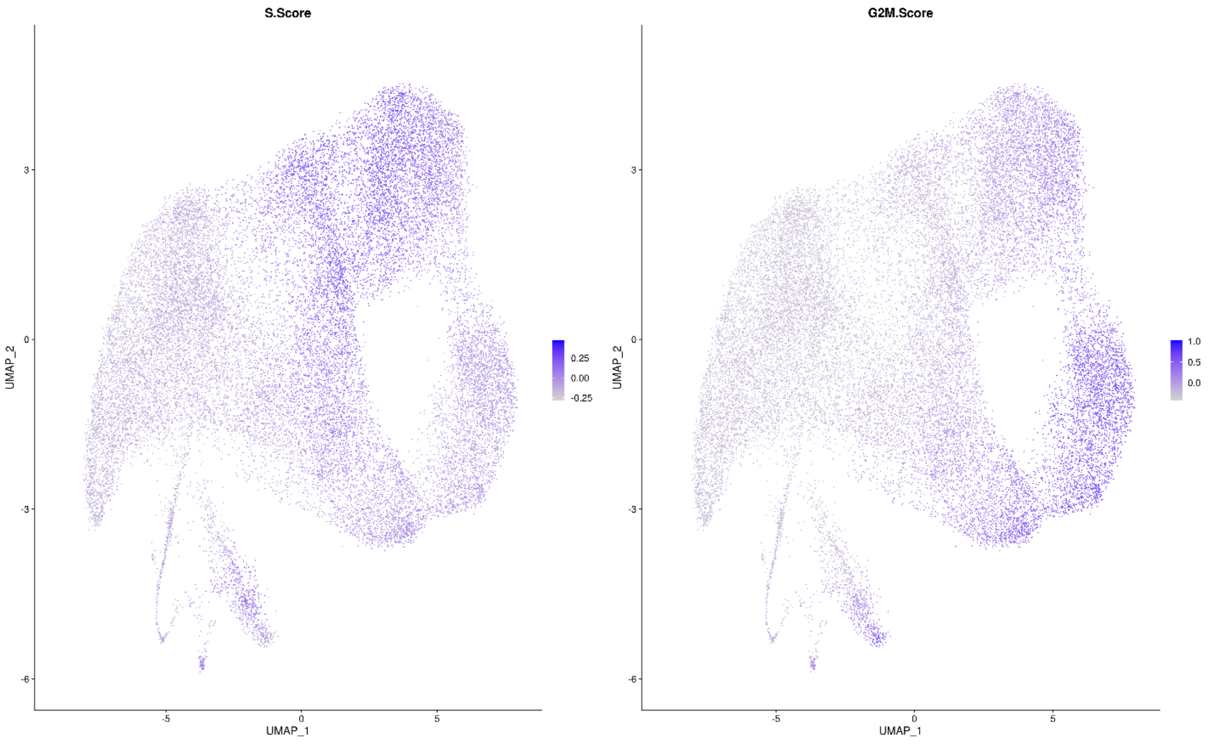

Figure S3. **Cell cycle scores based on the expression of G2/M and S phase markers in each cell.** The scores have been estimate using the Seurat package CellCycleScoring() function.

A anti-LRP1

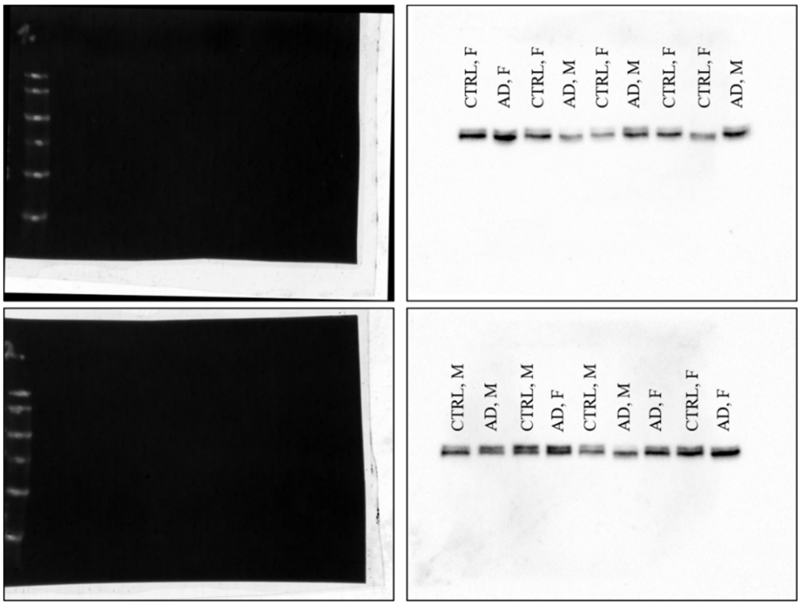

B anti- beta-actin

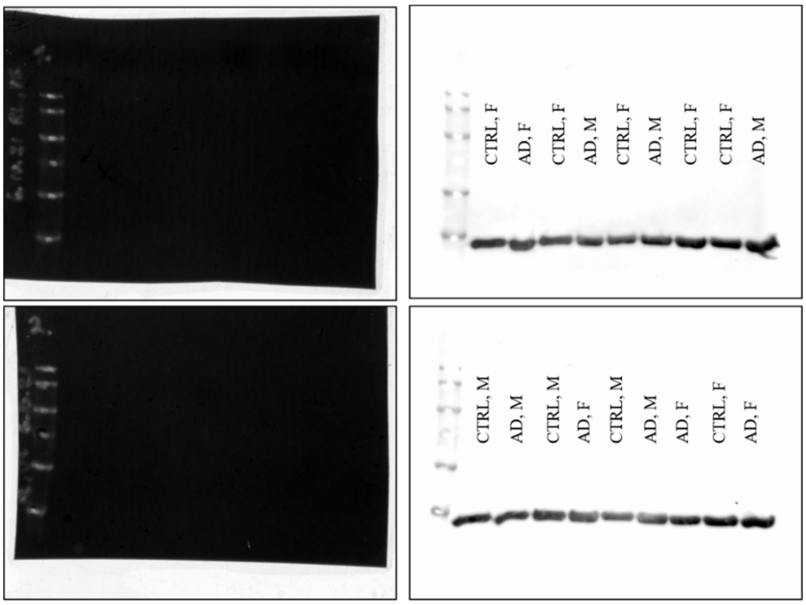

Figure S4. Uncropped immunoblot images for quantification of LRP1. Uncropped immunoblots for (A) LRP1 and (B) beta-actin. Prestained protein marker (PL00001, Proteintech, Rosemont, IL, USA) was used as a molecular weight marker on the gels/blots. CRTL, control. AD, Alzheimer's disease. F, female. M, male.

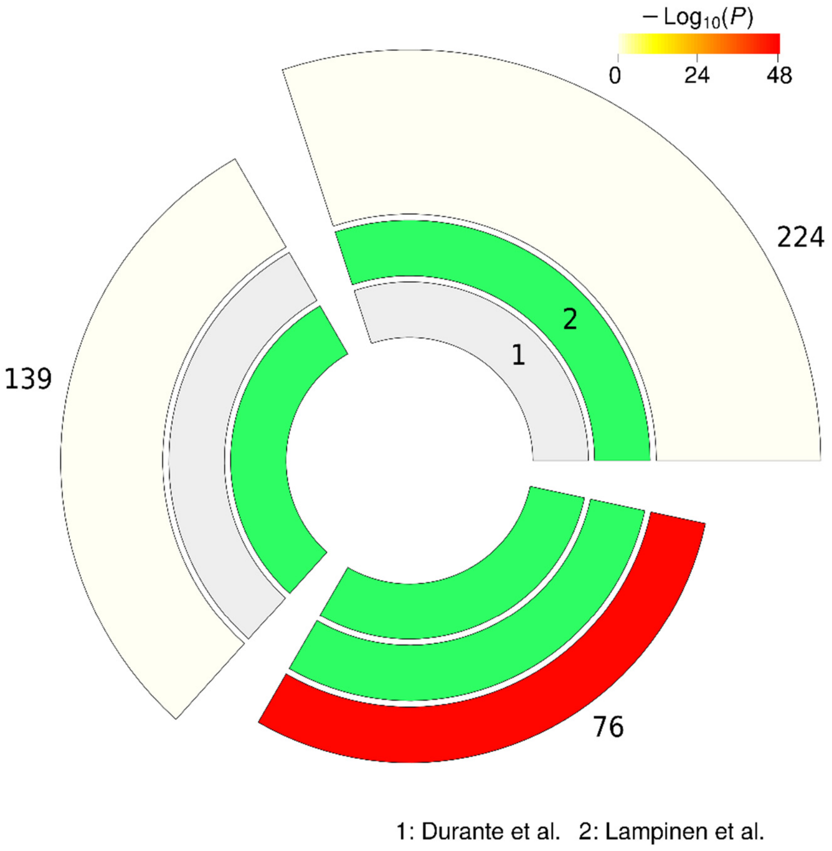

Figure S5. Statistical analysis of the overlapping pathways for GBC-like cells. Pie chart showing the significance of the intersection between pathways enriched from the GBC cell cluster markers of Durante et al. dataset and those identified as GBC-like cell clusters of our study. Significance is represented in the –log10(p value) scale. Green bands represent the two different lists of pathways and the values shown outside each piece of the chart represent the size of the overlapping pathways set.

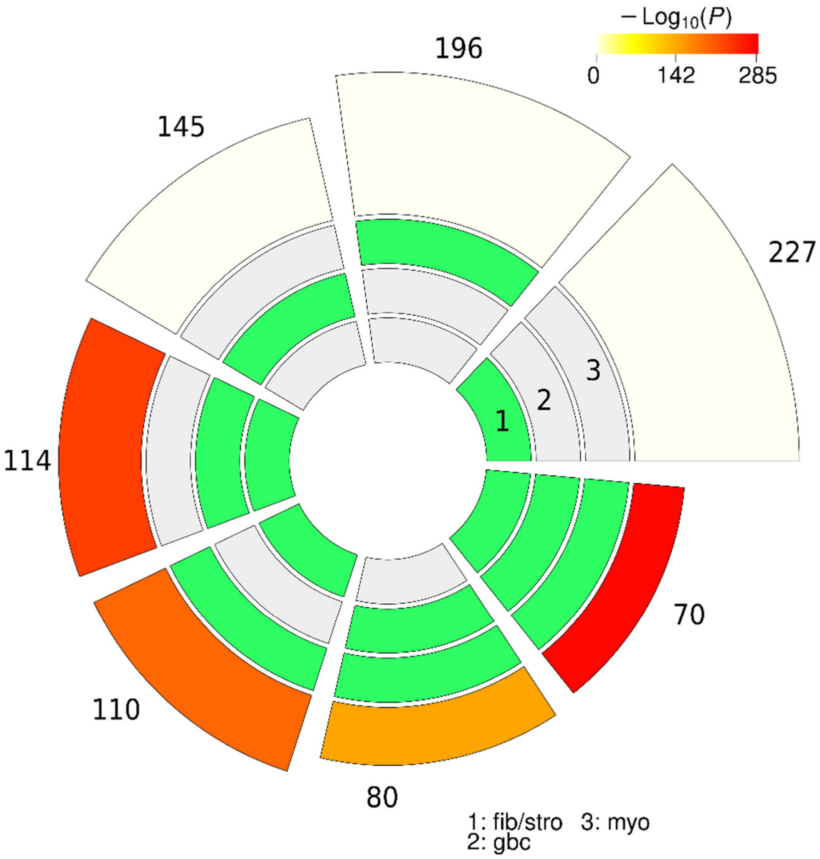

Figure S6. Statistical analysis of the overlapping DE genes by cell type. Pie chart showing the significance of the intersections between the three cell type DE genes, linked to AD, in the – log10(p value) scale. Green bands represent the different lists of DE genes and the values shown outside each piece of the chart represent the size of the overlapping genes set.

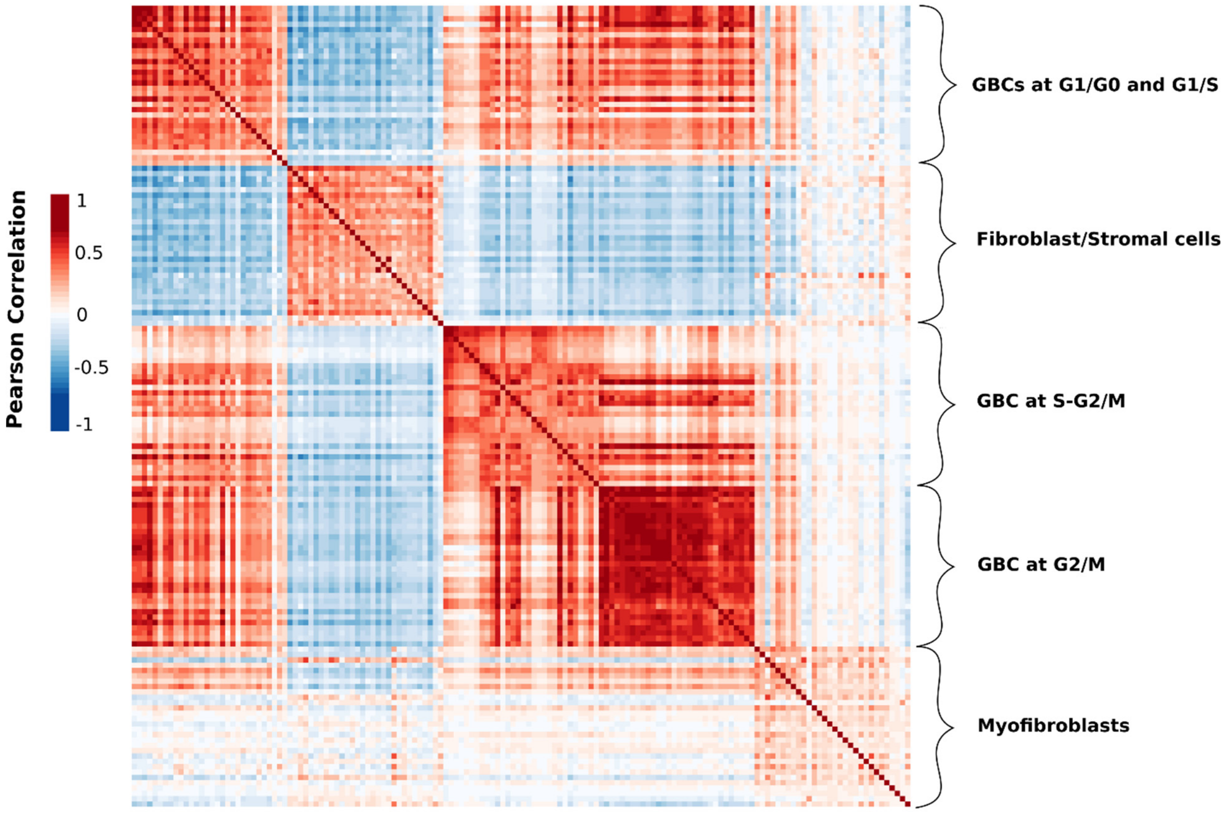

Figure S7. The heatmap representing a Pearson correlation matrix created using the marker genes used to identify the different cell types. The correlation matrix was computed using the rcorr() function of the R package *Hmisc.*

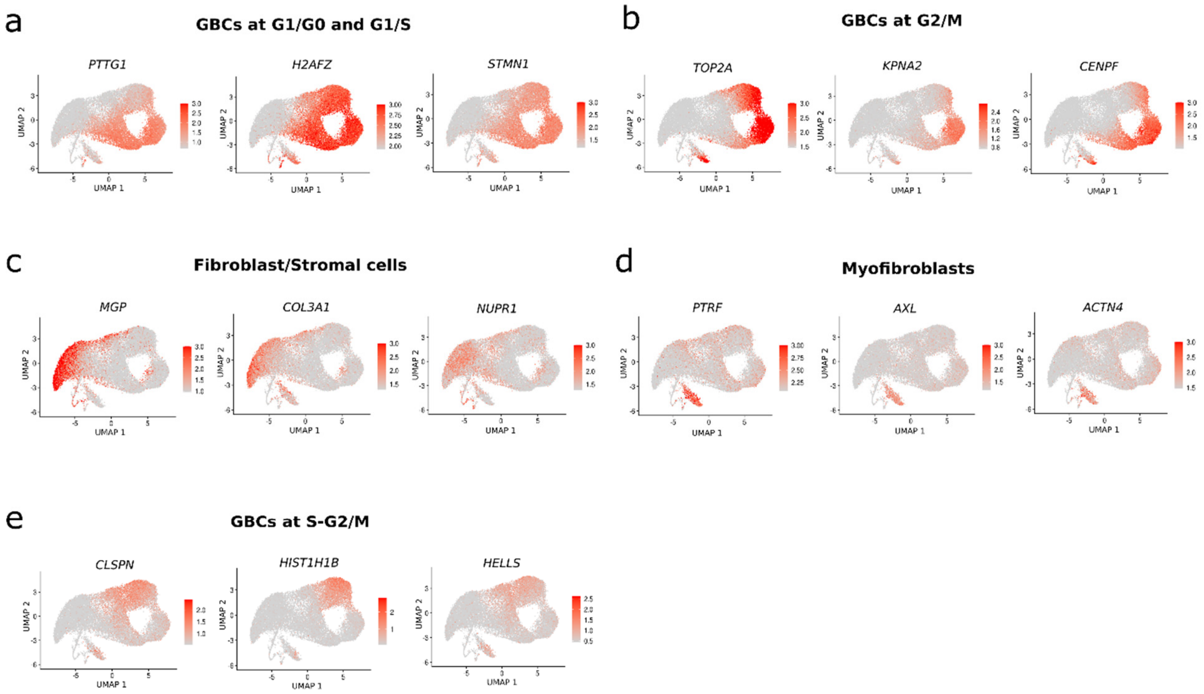

Figure S8. Feature plots for a subset of the 30 most up-regulated and significant differentially expressed genes between the clusters used for defining the cell populations. The plots (a-e) were produced using the FeaturePlot() function of Seurat package.

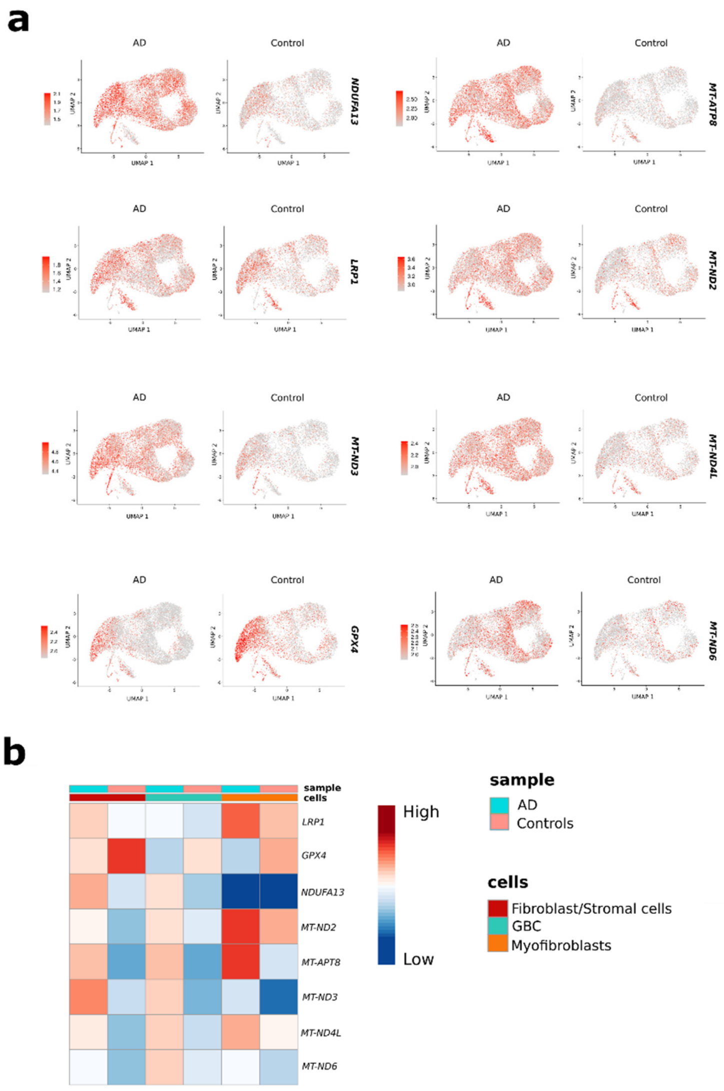

Figure S9. Feature plots (a) and heatmap (b) displaying cell-type specific alterations for a subset of DEGs showing transcriptional alterations between AD and control OM cells. The feature plots were produced using the FeaturePlot() function of Seurat package. The heatmap was achieved starting from the scaled average expression of the genes in the different cell types and conditions. The values range from 1.5 to –1.5.
